# Application of different patella height indices in patients undergoing total knee arthroplasty

**DOI:** 10.1186/s13018-017-0694-9

**Published:** 2017-12-12

**Authors:** Bin Xu, Wei-xing Xu, Di Lu, Hong-feng Sheng, Xin-wei Xu, Wei-guo Ding

**Affiliations:** Department of Orthopaedics, Tongde Hospital of Zhejiang Province, 234 Gu-cui Road, Hangzhou, 310012 People’s Republic of China

**Keywords:** Total knee arthroplasty, Radiographic measurement, Patella baja, Consistency test

## Abstract

**Background:**

One complication of total knee arthroplasty (TKA) is patella baja (PB). Patellar tendon shortening and joint line elevation are two main causes of PB. The purpose of this study was to determine the incidence of PB before and after TKA by measuring the patellar height and provide evidence for choosing a suitable index.

**Methods:**

In total, 256 consecutive patients who underwent primary TKA were included in this study. Radiographic measurements were performed; the Insall–Salvati (IS) index, modified IS (MIS) index, Blackburne–Peel (BP) index, and Caton–Deschamps (CD) index were computed; and the incidence of PB was calculated before and after the operation. The consistency between the IS and MIS indices and between the BP and CD indices was analyzed.

**Results:**

The preoperative incidence of true PB (TPB) and pseudo-PB (PPB) was 9.4 and 0.8%, respectively. The postoperative incidence of TPB and PPB was 10.2 and 9.0%, respectively. The consistency between the IS and MIS indices was moderate preoperatively (pre-kappa = 0.602) and postoperatively (post-kappa = 0.742). The consistency between the BP and CD indices was moderate preoperatively (pre-kappa = 0.742) and good postoperatively (post-kappa = 0.797).

**Conclusion:**

The incidence of PB, especially PPB, increased after TKA. The CD and BP indices are of greater importance for the diagnosis of PB after TKA. The MIS index is a better choice than the IS index to measure the length of the patellar tendon. To measure the height of the joint line, the BP index is better postoperatively and the CD index is better preoperatively.

## Background

Total knee arthroplasty (TKA) is an efficient treatment for end-stage knee osteoarthropathy [1]. However, both patellar tendon shortening and joint line elevation can result in patella baja (PB) [2, 3]. PB may cause anterior knee pain [3–6], limited range of motion (ROM) [3, 7], joint stiffness [8, 9], and prosthetic impact and abrasion [9, 10]. PB is divided into true PB (TPB) and pseudo-PB (PPB). TPB is caused by a short patellar tendon and results in pathological values for all indices, including the Insall–Salvati (IS) index [11], modified IS (MIS) index [12], Blackburne–Peel (BP) index [13], and Caton–Deschamps (CD) index [14]. The IS and MIS indices reflect the length of the patellar tendon, whereas the BP and CD indices reflect the distance between the distal pole of the patella and the tibial plateau. PPB is caused by joint line elevation without a short patellar tendon and results in pathological values for the CD and BP indices, whereas the IS and MIS indices stay within the normal range.

PB is not an uncommon complication, and its incidence has been reported as 34, 37, and 65% in different studies [15–17]. Postsurgical PB is mainly caused by patellar tendon shortening and joint line elevation [3, 18, 19]. However, which factor plays a more important role in the development of postsurgical PB remains unknown. The consistency between the IS and MIS indices and between the BP and CD indices is also unknown. While identification of the most important risk factor for postsurgical PB is a key to the prevention of PB after TKA, evaluation of the patellar height before TKA and determination of the optimal range of the above-mentioned indices to prevent PB are also important. How to combine these indices to evaluate the patellar height and diagnose PB remains unresolved.

The present study was performed to determine the consistency between the IS and MIS indices and between the BP and CD indices before and after TKA with the overall aim of identifying a suitable index for the diagnosis of PB. Through analysis of the aforementioned indices, we also investigated whether patellar tendon shortening or joint line elevation plays a more important role in the occurrence of postoperative PB.

## Methods

### Patient selection

From June 2015 to November 2016 at our institution, patients undergoing primary TKA for end-stage osteoarthropathy were included in this study. The inclusion criteria were the need for primary TKA, no extensor mechanism damage, no patellar dysontogenesis or fracture, and no history of trauma or surgery. The exclusion criteria were severe deformity, preoperative ROM of ≤ 90°, varus-valgus deformity of ≥ 15° [20], and severe bone defects of the femur or tibia.

### Operation protocol

All operations were performed with the patient under general anesthesia or combined spinal epidural anesthesia. All total knee prostheses were posterior stabilized cemented implants (P.F.C. Sigma; DePuy Orthopaedics, Raynham, MA, USA). We performed the operations with a midline incision and medial parapatellar approach. Distal femoral osteotomy was performed based on the extramedullary alignment system according to the knee physical valgus angle, which was measured preoperatively on anteroposterior weight-bearing radiographs. Tibia preparation was performed based on the intramedullary alignment system. The patella was routinely everted during the operation, patella reshaping was performed, and no patella resurfacing was carried out.

### Outcome measurement

**Fig. 1:**
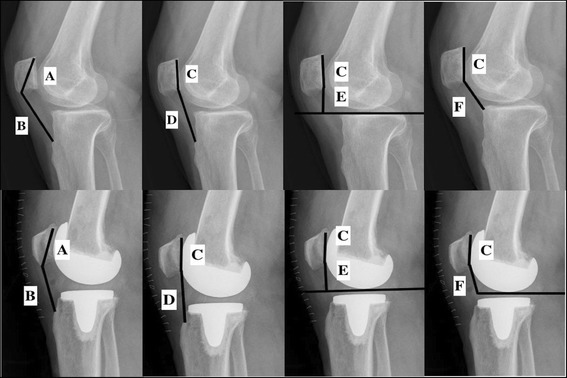
Knee joint measurements. A: diagonal length of patella. B: length of patellar tendon. C: length of patella articular surface. D: distance from patellar articular surface to tibial tuberosity. E: perpendicular height from the plateau line of the tibia to the lower end of the patellar articular surface. F: distance between patellar articular surface and anterior border of the tibial plateau. IS index = B/A, MIS index = D/C, BP index = E/C, CD index = F/C

**Fig. 2:**
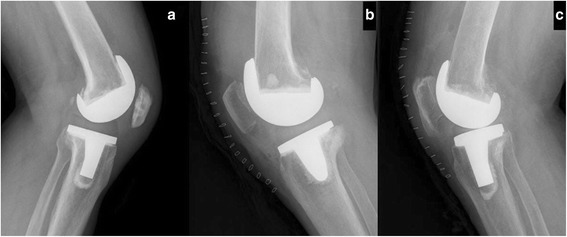
Patients with postoperative TPB, PPB, and Cyrano patella. **a** A 63-year-old man with postoperative TPB. IS = 0.65, MIS = 0.75, BP = 0.22, and CD = 0.30. **b** A 77-year-old man with postoperative PPB. IS = 0.90, MIS = 1.25, BP = 0.54, and CD = 0.56. **c** A 75-year-old woman with postoperative Cyrano patella. IS = 0.54, MIS = 1.28, BP = 0.58, and CD = 0.59

X-ray examinations of the knee joint in the 30° flexed position were performed before and after the operation. Software (syngo Imaging; Siemens, Munich, Germany) was employed to measure the diagonal length of the patella, the length of the patellar tendon, the length of the patella articular surface, the distance from the patella articular surface to the tibial tuberosity, the perpendicular height from the plateau line of the tibia to the lower end of the patella articular surface, and the distance between the patella articular surface and anterior border of the tibial plateau in the lateral view of the knee joint. The IS, MIS, BP, and CD indices were computed (see Fig. 1). TPB was diagnosed when the IS index was ≤ 0.8 or MIS index was ≤ 1.2, with a BP index of ≤ 0.54 or CD index of ≤ 0.60 (see Fig. 2a). PPB was diagnosed when the IS and MIS indices were within the normal range, while the BP index was ≤ 0.54 or CD index was ≤ 0.60 (see Fig. 2b) [2, 11–14, 21]. The incidence of TPB and PPB were calculated both preoperatively and postoperatively. To minimize interobserver variation, two blind observers performed the measurements and calculations in the radiograph. The mean of the results from the two observers were used for the final analysis.

### Statistical analysis

All data were analyzed using SPSS version 19.0 (IBM Corp., Armonk, NY, USA). The kappa test was employed to analyze the consistency between the IS and MIS indices and between the BP and CD indices. The consistency was poor when kappa ≤ 0.04, moderate when 0.4 < kappa < 0.75, and good when kappa ≥ 0.75.

## Results

### General patient characteristics

Among the 256 patients included in this study, 42 were men and 214 were women. Their average age was 64.56 ± 7.98 years, and their average body mass index was 25.77 ± 3.56 kg/m^2^.

### Incidence of PB

Preoperatively, the incidence of TPB and PPB was 9.4% (24 cases) and 0.8% (2 cases), respectively. The positive rate of the IS index was 7.8% (20 cases), MIS index was 5.9% (15 cases), BP index was 3.1% (8 cases), and CD index was 3.1% (8 cases). Postoperatively, the incidence of TPB and PPB was 10.2% (26 cases) and 9.0% (23 cases), respectively. The positive rate of the IS index was 8.2% (21 cases), MIS index was 9.0% (23 cases), BP index was 15.2% (39 cases), and CD index was 14.1% (36 cases).

The consistency between the IS and MIS indices was moderate both preoperatively (pre-kappa = 0.602) and postoperatively (post-kappa = 0.742), but was greater postoperatively than preoperatively. The consistency between the BP and CD indices was moderate preoperatively (pre-kappa = 0.742) and good postoperatively (post-kappa = 0.797) (see Table 1).

**Table 1:** Number of positive and negative cases of all indices before and after total knee arthroplasty

Index	Preoperation	Postoperation
IS (+)	20	21
MIS (+)	11	18
MIS (−)	9	5
IS (−)	236	235
MIS (+)	4	5
MIS (−)	232	230
MIS (+)/(−)	15/241	23/233
BP (+)	8	39
CD (+)	6	31
CD (−)	2	8
BP (−)	248	217
CD (+)	2	5
CD (−)	246	212
CD (+)/(−)	8/248	36/220

*IS* Insall–Salvati, *MIS* modified Insall–Salvati, *BP* Blackburne–Peel, *CD* Caton–Deschamps, *IS (+)* number of cases of positive IS index, *IS (−)* number of cases of negative IS index, *MIS (+)* number of cases of positive MIS index, *MIS (−)* number of cases of negative MIS index, *BP (+)* number of cases of positive BP index, *BP (−)* number of cases of negative BP index, *CD (+)* number of cases of positive CD index, *CD (−)* number of cases of negative CD index

### Changes in parameters

Compared with the preoperative value, the MIS index decreased after TKA (preoperative: 1.61 ± 0.22, postoperative: 1.51 ± 0.21; *P* < 0.0001), reflecting patellar tendon shortening. The BP index (preoperative: 0.86 ± 0.16, postoperative: 0.74 ± 0.14; *P* < 0.0001) and CD index (preoperative: 0.93 ± 0.15, postoperative: 0.78 ± 0.12; *P* < 0.0001) decreased after TKA, reflecting joint elevation. The IS index (preoperative: 1.07 ± 0.19, postoperative: 1.07 ± 0.17; *P* = 1) changed little after TKA because the patellar tendon was shortened when the diagonal length of the patella was reduced during patellar reshaping (see Table 2).

**Table 2:** Comparison of all indices before and after total knee arthroplasty

	Preoperation	Postoperation	*P*
IS index	1.07 ± 0.19	1.07 ± 0.17	1
MIS index	1.61 ± 0.22	1.51 ± 0.21	< 0.0001
BP index	0.86 ± 0.16	0.74 ± 0.14	< 0.0001
CD index	0.93 ± 0.15	0.78 ± 0.12	< 0.0001

*IS* Insall–Salvati, *MIS* modified Insall–Salvati, *BP* Blackburne–Peel, *CD* Caton–Deschamps

## Discussion

Patellar tendon shortening and joint line elevation are two main causes of PB. Shortening of the patellar tendon is related to the reconstruction of the anterior cruciate ligament [18, 22, 23], patellar fracture [24], high tibial and distal femoral osteotomies [25, 26], and other factors. Although the standard medial parapatellar approach can provide a good operative field through patellar eversion [27–29], there is a risk of injuring the patellar tendon during the operation or even stripping the tendon from the tibial tubercle [30–32]. Additionally, the decreased strength of the quadriceps femoris muscle may cause contracture of the patellar tendon [32]. Floren et al. [17] reported that 37% of patients had patellar tendon shortening of > 5% through the standard medial parapatellar approach and that 14% of patients had shortening of > 10%. Koshino et al. [15] reported that 65% of patients had patellar tendon shortening of > 10%, while Weale et al. [16] reported a rate of 34%. Joint line elevation may be the result of excessive soft tissue release, excessive distal femoral osteotomy [33, 34], insufficient tibial plateau osteotomy [35], and a large insert thickness [2]. Joint line elevation caused by TKA is not uncommon; the incidence can reach 40% after primary TKA [13, 36–38]. Kawamura and Bourne [39] reported a mean joint line elevation of 3.5 mm, Sabbioni et al. [40] reported 4.0 mm, and Scuderi and Insall [41] reported 5.6 mm.

When patellar tendon shortening exceeds 10%, a negative correlation exists between the shortening and ROM of the knee and between the patellar position and knee function [33]. Joint line elevation can change the knee kinematics [35, 37] and result in distal shift of the patella, increasing both the pressure on the patella when the knee is flexed and the tension on the soft tissue [42, 43]. Joint line elevation after TKA is likely to result in anterior knee pain, decreased ROM, decreased knee function, and patella component impingement on the tibia or polyethylene [21, 33, 35]. Estupinan et al. [44] claimed that a shift in the joint line itself induces wear of the polyethylene, and Rand [43] suggested that greater proximal joint line shifts correspond to more distal patellar movement compared with the preoperative position. Partington et al. [45] reported that joint line elevation of > 8 mm yields inferior clinical results, and Porteous et al. [46] found that joint line elevation of > 5 mm worsened clinical outcomes. Emodi et al. [47] suggested that even mild joint line elevation of < 2 mm alters the transmission of myogenic strength in the quadriceps and increases patellofemoral pressure forces.

In the present study, the preoperative incidence of TPB was higher than PPB, indicating that patellar tendon shortening was the main reason. The MIS, BP, and CD indices decreased after the operation, indicating that the incidence of PB increased and that both patellar tendon shortening and joint line elevation played a role in this change. Although the IS index changed little after the operation, the incidence of PB diagnosed by the IS index increased when false-positive cases were excluded. Thus, the incidence of PB increased after TKA. In addition, the incidence of TPB increased from 9.4% (24 cases) to 10.2% (26 cases) after TKA, while the incidence of PPB increased from 0.8% (2 cases) to 9.0% (23 cases). Because the increase of PPB was higher, the PB caused by TKA was more strongly associated with joint line elevation. Restoration of the proper joint line is therefore a critical factor for successful TKA, indicating that evaluation of the patellar height before and after TKA is important.

Performing X-ray examinations of the knee joint is necessary before and after TKA. The IS, MIS, BP, and CD indices are simple and commonly used measures with which to evaluate the patellar height, and they can be measured on a roentgenogram. Although measurements using computed tomography or magnetic resonance imaging may be more accurate, we prefer using roentgenography because it has the following advantages. The IS and MIS indices are used to evaluate the length of the patellar tendon, and both of these indices use the tibial tuberosity as a landmark. The BP and CD indices are used to evaluate the height of the joint line, and both of these indices use the tibial plateau as a landmark. The IS and MIS indices can detect TPB, but they cannot detect PPB caused by joint line elevation. The BP and CD indices are important measures with which to detect PPB because they synthetically consider the length of the patellar tendon and the height of the joint line. The combination of these two indices will help to more comprehensively evaluate PB and provide a basis for detecting TPB or PPB.

The indices employed to evaluate the patellar height involve a ratio between two measurements. One measures the patellar length, and the other measures the distance between the patella and the tibia. A ratio can compensate for variations in patients’ height, while an absolute length cannot. Although numerous indices are available to evaluate the patellar height, no single index is perfect. The IS index is more likely to be influenced by the shape of the patella. The incidence of PB will be overestimated in patients with a Cyrano patella (i.e., patella with a long nonarticulating inferior pole) (see Fig. 2c) [48]. The MIS index is not influenced by the length of the inferior pole, and its use will decrease the rate of false-positive cases. The consistency between the IS and MIS indices was moderate preoperatively but better postoperatively because false-positive cases of the IS index were present preoperatively and eliminated through patella reshaping. Thus, the MIS index is more suitable for evaluation of the length of the patellar tendon preoperatively, and both the IS and MIS indices are suitable for evaluation of the length of the patellar tendon postoperatively. The BP index requires a line to be drawn along the tibial plateau, which is inconvenient to do on a roentgenogram, and the overlap of the medial and lateral plateaus must be perfect for accurate placement of the line. However, placement of a perfect line on the roentgenogram rarely occurs in every case. Although the preoperative consistency between the BP and CD indices was relatively good in our study, the CD index is a better choice for evaluation of the distance between the distal pole of the patella and the tibial plateau. Locating the anterior border of the insert is more difficult postoperatively because it is invisible on the roentgenogram. Therefore, postoperatively, the BP index is a better choice for evaluation of the distance between the distal pole of the patella and the tibial plateau; even the postoperative consistency was good.

Seo et al. [49] proposed an effective method with which to prevent joint line elevation through application of an additional metal block to reduce excess resection of the distal femur, successfully decreasing the incidence of PPB from 92 to 11%. However, they chose patients who had a ≤ 15-mm perpendicular height from the plateau line of the tibia to the lower end of the patellar articular surface. As mentioned above, an absolute length cannot compensate for the variations in patients’ height. Determination of the optimal range of a proper index is therefore needed to prevent PB. In this study, we provide evidence for choosing the proper index with which to evaluate the patellar height and changing different indices after TKA, thus establishing a basis for finding such a range.

This study had several limitations. The radiological measurements obtained by roentgenography may have been influenced by the presence of severe osteophytes or inaccurate patient positioning (e.g., deviation from the 30° flexed position or image overlays). Because the positive rate of PB was low, the number of positive cases was small in our study. This study simply demonstrated the radiologic properties before and after TKA without clinical data. Further investigations of clinical outcomes among patients with TPB, PPB, or a normal patella are needed.

## Conclusion

In conclusion, the IS and MIS indices can be used to detect TPB. The MIS index is more suitable for preoperative evaluation of the length of the patellar tendon. Although both the IS index and the MIS index are suitable for evaluating the length of the patellar tendon postoperatively, the MIS index is a better choice. Along with the IS and MIS indices, the BP and CD indices are important in detecting PPB. Although both the BP and CD indices are suitable for evaluating the distance between the distal pole of the patella and the tibial plateau, the CD index is a better choice preoperatively and the BP index is a better choice postoperatively. PB caused by TKA is more strongly associated with joint line elevation, which means that the CD and BP indices are of greater importance for the diagnosis of PB after TKA.

## Abbreviations

BP: Blackburne–Peel
CD: Caton–Deschamps
IS: Insall–Salvati
MIS: Modified Insall–Salvati
PB: Patella baja
PPB: Pseudo-patella baja
ROM: Range of motion
TKA: Total knee arthroplasty
TPB: True patella baja
